# Effects of Qili Qiangxin Capsule on AQP2, V2R, and AT1R in Rats with Chronic Heart Failure

**DOI:** 10.1155/2015/639450

**Published:** 2015-05-17

**Authors:** Xiangning Cui, Jian Zhang, Yubo Li, Yuxiu Sun, Jian Cao, Mingjing Zhao, Yizhou Zhao, Xin Zhao, Yaoyao He, Anbang Han

**Affiliations:** ^1^Department of Cardiology, Guang'anmen Hospital, China Academy of Chinese Medical Sciences, Beijing 100053, China; ^2^Beijing University of Chinese Medicine, Beijing 100029, China; ^3^Institute of Basic Theory, China Academy of Traditional Chinese Medicine, Beijing 100700, China; ^4^The Key Laboratory of Chinese Internal Medicine of the Ministry of Education, Dongzhimen Hospital Affiliated to Beijing University of Chinese Medicine, Beijing 100700, China

## Abstract

Qili qiangxin capsule (QL), a traditional Chinese herbal compound, has been proved to be effective and safe for the treatment of chronic heart failure (CHF). Upregulation of aquaporin-2 (AQP2) accounts for the water retention in CHF. The aim of the present study was to evaluate the effects of QL on the expression of AQP2 in rats with CHF induced by acute myocardial infarction and to investigate the underlying mechanisms. The urine output of all rats was quantified and collected every day at the first week and the 4th week after administration of QL or Valsartan. The expression of AQP2, vasopressin type 2 receptor (V2R), and angiotensin II type 1 receptor (AT1R) were examined after treatment for 4 weeks. Urinary output increased significantly after administration of QL. Importantly, the protein expression of AQP2 and AQP2 phosphorylated at serine 256 (pS256-AQP2) was downregulated after administration of QL and Valsartan to CHF rats. Furthermore, QL reduced plasma arginine vasopressin (AVP) and angiotensin II (AngII) level and downregulated V2R and AT1R protein expression. Thus, QL exerts its diuretic effect and improves cardiac function in CHF rats by reversing the increases in both AQP2 and pS256-AQP2 expression. The possible mechanisms may involve inhibition of V2R and AT1R.

## 1. Introduction

Chronic heart failure (CHF) is now recognized as a major and escalating public health problem. The prevalence of CHF is 1-2% and appears to be increasing, in part because of ageing of the population [1]. It has an annual hospitalisation rate of 2% with subsequent 1-year mortality of 30% [2]. CHF has become a major public health burden in the world that is associated with high morbidity, mortality, and cost [3, 4].

Water retention, the hallmark feature of HF, not only causes signs and symptoms of congestion, but also impacts myocardial remodeling and HF progression [5]. Thus, improving congestion is a cornerstone of HF management. Although loop diuretics are among the most commonly prescribed drugs in this setting, there are some adverse drug reactions associated with their use. Moreover, up to 30% of the patients with decompensated HF present with loop-diuretic resistance [6]. Therefore, novel and safe therapeutic approaches for the treatment of congestion have been of interest in recent research.

Water retention in HF is primarily due to the defects in renal handling of sodium and water, resulting in the increasing of water reabsorption. A number of studies have proved that aquaporin-2 (AQP2), a channel that is exclusively selective for water molecules and never allows permeation of ions or other small molecules, primarily expressed in the collecting duct of the kidney, plays an important role in the regulation of urinary concentration and control of fluid and electrolyte homeostasis. In rat models of CHF, there was a significant increase in AQP2 mRNA and protein levels and the targeting of AQP2 to the apical membrane, both of which elevate the water permeability of the collecting duct cells, resulting in the promotion of water reabsorption from the urinary tubule [7, 8]. Therefore, AQP2 impairment is an important cause of water retention that exacerbates the prognosis of congestive heart failure, which is now attracting considerable attention as a novel therapeutic target for water balance disorders which commonly occur in CHF.

The renin-angiotensin system and the nonosmotic release of arginine vasopressin (AVP) are increased in cardiac failure. Recent experimental evidence suggested that, in addition to the interaction between angiotensin AT1 and AVP V1 receptors on systemic and renal vasculature, there may be an important interaction between AVP and angiotensin II (AngII) in the regulation of AQP2 [9–11].

Qili qiangxin capsule is a traditional Chinese medicine that was approved by China Food and Drug Administration for the treatment of heart failure in 2004. Recently, a double-blind, multicenter, placebo-controlled, prospective, randomized clinical trial of qili qiangxin capsules in more than 500 patients with chronic heart failure was finished; it has proved qili qiangxin's efficacy and safety [12]. Previous studies showed that qili qiangxin capsule can improve cardiac function, inhibit the development of cardiac hypertrophy and remodeling, and regulate the inflammatory responses [13–15]. QL also inhibited *I*_Ca-L_, *I*_Na_, and multi-K^+^ channels of ventricular myocytes [16, 17]. QL has been shown to have protective effects on energy metabolism and myocardial mitochondria in pressure overload heart failure rats [18]. However, little is known about whether qili qiangxin has a role in improving water retention in CHF. Therefore, using a postmyocardial infarction heart failure model, the present study was carried out to determine (1) whether QL can reduce volume overload and improve cardiac function in the late phase of infarction, (2) whether AQP2 and pS256-AQP2 are involved in the effects of QL, and (3) whether QL can regulate AVP-V2R-AQP2 and AngII-AT1R-AQP2 signaling in the renal medulla. The effects of QL were also compared with Valsartan, a commonly used AT1R blocker in clinical practice, which is known to have cardioprotective effects in the treatment of HF.

## 2. Materials and Methods

### 2.1. Vegetal Material

Qili qiangxin consists of Ginseng, Radix Astragali, Aconite Root, Salvia miltiorrhiza, Semen Lepidii Apetali, Cortex Periplocae Sepii Radicis, Rhizoma Alismatis, Carthamus tinctorius, Polygonatum Odorati, Seasoned Orange Peel, and Ramulus Cinnamomi (Yiling Pharmaceutical Corporation, Shijiazhuang, China). The drug powder was dissolved with sterile water at the concentration of 0.1 g/mL Qili qiangxin was prepared for the study. Valsartan (batch number X1428) was manufactured by Beijing Novartis Pharmaceutical Co. Ltd and dissolved with sterile water.

### 2.2. Animal Model and Administration

Normal male Sprague-Dawley rats (body weight 220~250 g) were provided by Beijing Vital River Laboratory Animal Technology Co. Ltd (Animal license number: SCXK (Beijing) 2012-0001). The animals were fed with standard diet and water and were subject to a 12 h light and 12 h dark cycle, a temperature of 20 ± 2°C, and a humidity of 50 ± 2%. All animal experimental protocols were approved by Animal Care and Use Committee of Beijing University of Chinese Medicine and complied with laboratory animal management and use regulations. HF was induced by myocardial infarction following ligation of the left anterior descending artery (LAD). Sodium pentobarbital 1% (50 mg/kg) was administered by intraperitoneal injection. The procedures performed included endotracheal intubation, ventilator positive pressure ventilation, preoperative recording of 12-lead ECG, local skin disinfection, chest opening, thoracotomy device setup, and opening of the pericardium, the pulmonary cone, and the left atrial appendage 2~3 mm from the bottom of the left anterior descending coronary artery ligation. For the rats assigned to the sham group, the same operation was performed without ligation of the left coronary artery. Twelve-lead ECG was recorded after the experiments. MI rats were fed normally for 4 weeks. According to transthoracic echocardiography results (Table 1), the survival rats were randomly assigned to the following groups: Model group (CHF, *n* = 12), Sham group (Sham, *n* = 10), QL group (QL, *n* = 9), and Valsartan group (Valsartan, *n* = 9). QL 1.0 g/kg and Valsartan 10 mg/kg were administered, respectively, by gavage once a day during the 4 weeks. Equal volume of distilled water was used for model and sham group. The urine of all rats was collected for 24 h. The urine volume was determined daily.

### 2.3. Transthoracic Echocardiography Measurements

*A noninvasive transthoracic* echocardiography method was used to evaluate the morphology and function of left ventricle. Echocardiography was performed in anesthetized animals. It consisted of a two-dimensional mode, that is, time-motion (TM) mode and blood flow measurements in pulsed Doppler mode.

### 2.4. Sample Preparation and Histological Examination

At the end of 4 weeks after treatment, rats were anesthetized. A blood sample from the aorta was centrifuged and plasma was collected for AVP and AngII measurements. Rat hearts were rapidly removed and rinsed with cold physiological saline, and water was adsorbed by filter paper. The excess tissues around the hearts were removed, and hearts were weighed and then were fixed in 4% paraformaldehyde and embedded in paraffin. Rat heart samples were cut into transverse sections and stained with haematoxylin and eosin (H&E). Weight index was calculated using the following formula: index = heart weight (HW)/body weight (BW).

### 2.5. Preparation of Tissue for Immunocytochemistry

Rats were perfused and fixed with 4% PFA after anesthetizing with 10% chloral hydrate. The kidneys were stripped off and soaked in 4% PFA for 14 h. The fixed kidneys were dehydrated by 30% sucrose for 24 h and embedded in OCT medium after cryoprotection function. Frozen kidneys were cut into 30 *μ*m coronal sections prelocated at caudate putamen by a refrigerated microtome. Endogenous peroxidase activity was blocked with 0.1% H_2_O_2_ in absolute methanol for 10 min at room temperature. To expose antigens, kidney sections were boiled in a target retrieval solution (1 mmol/l Tris, pH 9.0 with 0.5 mM EGTA) for 10 min. After cooling, incubate the tissue sections in 50 mM NH_4_Cl in PBS for 30 min to prevent nonspecific binding, followed by blocking in PBS containing 1% BSA, 0.05% saponin, and 0.2% gelatin. Sections were incubated with primary antibodies diluted in PBS with 0.1% BSA and 0.3% Triton X-100 overnight at 4°C. After being washed for 3~10 min with PBS supplemented with 0.1% BSA, 0.05% saponin, and 0.2% gelatin, the sections were incubated with HRP-conjugated secondary antibody for 1 h at room temperature. After rinsing with PBS wash buffer, the sites of antibody-antigen reactions were visualized with 0.05% 3, 3′-diaminobenzidine tetrachloride dissolved in distilled water with 0.1% H_2_O_2_. Then light microscopy was carried out.

### 2.6. Western Blot Analysis

All animals were euthanized after 4 weeks of drug administration, and their hearts were immediately harvested and stored in liquid nitrogen until Western blot analyses were performed. The following antibodies were used: rabbit polyclonal Anti-Aquaporin 2 (1 : 2000, ABcam, Inc.), rabbit polyclonal Anti-Aquaporin 2 (phospho S256) (1 : 1000, ABcam, Inc.), rabbit polyclonal Anti-V2R (1 : 2000, ABcam, Inc.), and rabbit polyclonal Anti-AT1R (1 : 2000, ABcam, Inc.). Proteins were separated by 10% SDS-PAGE and transferred to nitrocellulose membranes, which were then incubated with antibodies at 4°C. The membranes were further incubated with horseradish peroxidase-conjugated anti-rabbit IgG (1 : 2,000) for 2 hours at room temperature. ECL visualisation was performed and the Gene Gnome Gel Imaging System (Syngene Co.) was used to capture the resulting images. Image J (NIH image, Bethesda, MD) was used to analysis the gel images.

### 2.7. Statistical Methods

All experimental data were presented as mean ± SD, single factor analysis of variance (ANOVA) was performed with the statistical software SPSS17.0, Dunnett's T3 was used for unequal variances, and A probability of *P* < 0.05 was considered as statistically significant.

## 3. Result

### 3.1. Effects of QL on Urinary Output at 1 or 4 Weeks after Treatment

**Figure 1:**
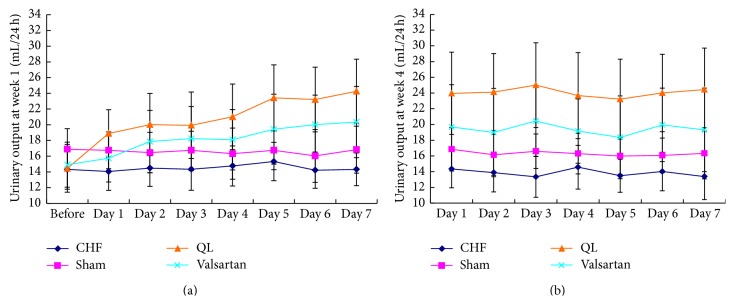
Both QL and Valsartan increased urinary output in CHF rats. (a) Urinary output was significantly increased in the rats treated with QL (*n* = 9) for 1 week compared with the CHF group (*n* = 12). Urinary output was slightly increased in Valsartan treatment group (*n* = 9). (b) Urinary output was increased in the rats treated with QL or Valsartan at the 4th week compared with the CHF group.

To determine whether QL has diuretic effect, we measured urinary volume in rats in every group before and after treatment with QL or Valsartan for 1 or 4 weeks. We found that urinary volume was markedly increased after treatment with QL from week 1 to week 4. However urinary volume was slightly increased in Valsartan treatment group (Figures 1(a) and 1(b)). Therefore, QL has a diuretic action, and can reduce extracellular volume in CHF rats by increasing urinary volume.

### 3.2. Effects of QL on Survival Rate and Heart Weight/Body Weight Ratio at the 4th Week after Treatment

**Figure 2:**
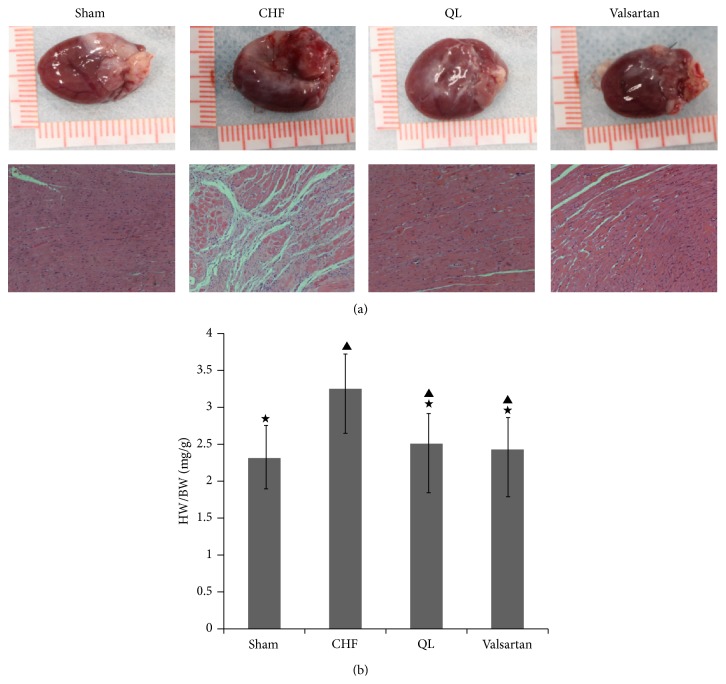
Heart preparation and pathological sections from normal and CHF rats. (a) Heart preparations [top (1~4)] and pathological sections [bottom (5~8)] from the sham group, the CHF group, the QL group, and the Valsartan group. (b) HW (heart weight)/BW (body weight) ratios in the sham group (*n* = 10), the CHF group (*n* = 11), the QL group (*n* = 8), and the Valsartan group (*n* = 8). (^★^ *P* < 0.05, versus the CHF group, ^▲^ *P* < 0.05, versus the sham group).

After treatment for 4 weeks, deaths had occurred, and the survival rates of the sham group (*n* = 10), the CHF group (*n* = 11), the QL group (*n* = 8), and the Valsartan group (*n* = 8) were therefore 100%, 92%, 89%, and 89%, respectively. As shown in Figures 2(a) and 2(b), long-term treatment with either QL or Valsartan significantly reduced the heart weight/body weight ratio (*P* < 0.05).

### 3.3. Posttreatment Assessment of Cardiac Structure and Function by Echocardiography

**Figure 3:**
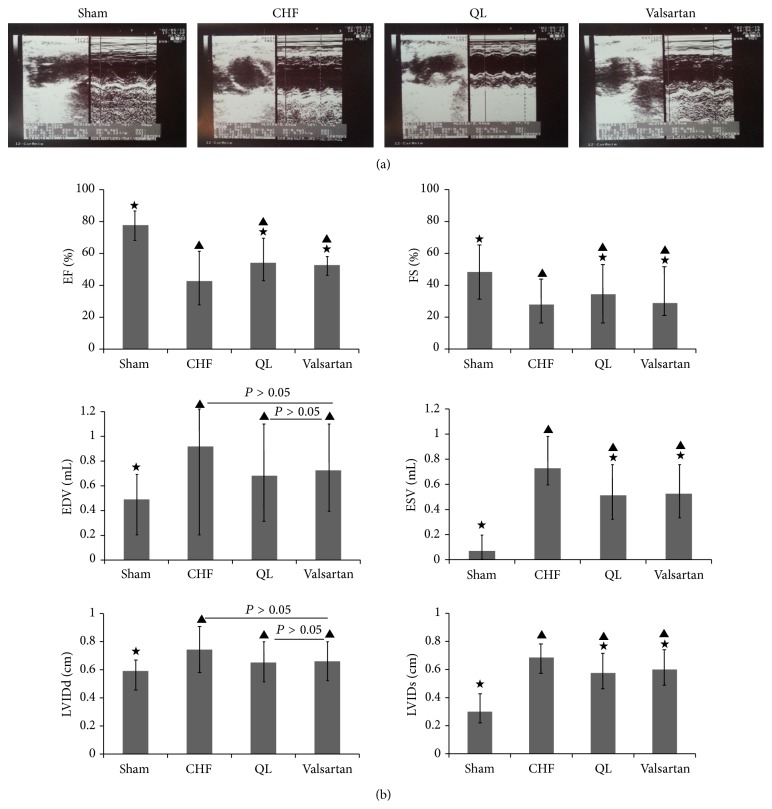
Typical echocardiography images from the sham group, the CHF group, the QL group, and the Valsartan group. At the 4th week of QL and Valsartan administration, cardiac structure and function were measured in each group by echocardiography. We evaluated cardiac systolic and diastolic functions by measuring the following variables: ejection fraction (EF), fractional shortening (FS), end-diastolic volume (EDV), end-systolic volume (ESV), left ventricular end-diastolic dimension (LVIDd), and left ventricular end-systolic dimension (LVIDs). Treatment with either QL or Valsartan improved systolic function. (^★^ *P* < 0.05, versus the CHF group, ^▲^ *P* < 0.05, versus the sham group).

Compared with the CHF group (*n* = 11), the ejection fraction (EF) and fractional shortening (FS) measurements were elevated in the QL group (*n* = 8) and the Valsartan group (*n* = 8) (*P* < 0.05), while the end-systolic volume (ESV) and left ventricular end-systolic dimension (LVIDs) measurements were lowered (*P* < 0.05). Although the measurements obtained for end-diastolic volume (EDV) and left ventricular end-diastolic dimension (LVIDd) displayed a decreasing trend in both the QL group and the Valsartan group versus the CHF group, the difference was not statistically significant (*P* > 0.05). Compared with those of the sham group, the EF and FS measurements obtained from the CHF group, the QL group, and the Valsartan group were reduced (*P* < 0.05), while the ESV and LVIDs measurements were increased (*P* < 0.05) (Figures 3(a) and 3(b)).

### 3.4. Effects of QL on the Expression of Total AQP2 in CHF Rats

**Figure 4:**
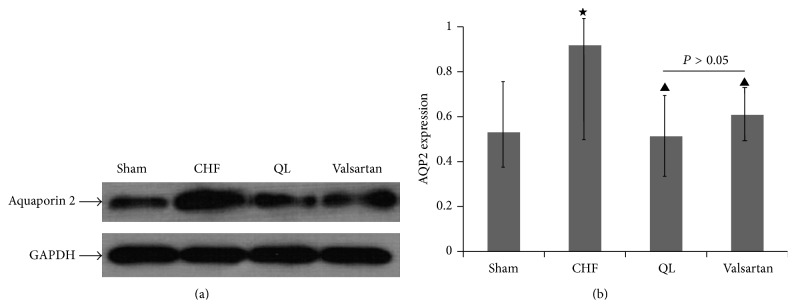
QL reduces renal AQP2 expression in CHF rats. (a) Representative images show that treatment with QL markedly attenuated AQP2 protein expression in CHF rats. (b) Densitometric analysis of the data demonstrated a significantly decreased AQP2 protein abundance in QL and Valsartan treatment rats compared with CHF rats. (^★^ *P* < 0.05, versus the sham group, ^▲^ *P* < 0.05, versus the CHF group).

Semiquantitative immunoblotting (Figures 4(a) and 4(b)) revealed that AQP2 protein abundance was significantly increased in the CHF rats compared with sham-operated rats (*P* < 0.05). Compared with the CHF group, the AQP2 protein abundance was reduced in both the QL group and the Valsartan group (*P* < 0.05), while no significant differences were observed in AQP2 protein abundance between QL group and Valsartan group (*P* > 0.05).

**Figure 5:**
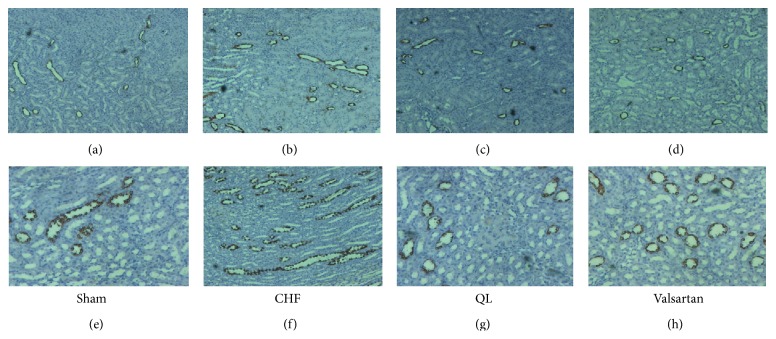
Immunohistochemistry for AQP2 expression in the groups: sham ((a) and (e)), CHF ((b) and (f)), QL ((c) and (g)), and valsartan ((d) and (h)) at different magnifications. AQP2 expression was detected in principal cells in the collecting ducts, and labeling was much stronger in rats with CHF than in sham rats; labeling was much weaker in QL and Valsartan rats.

Consistent with this, immunohistochemical analysis (Figure 5) showed a much stronger labeling of anti-AQP2 antibody that was detected in principal cells in the collecting ducts in the CHF rats compared with sham-operated control rats, and labeling was much weaker in rats of QL group and the Valsartan group.

### 3.5. Effects of QL on the Expression of pS256-AQP2 in HF Rats

**Figure 6:**
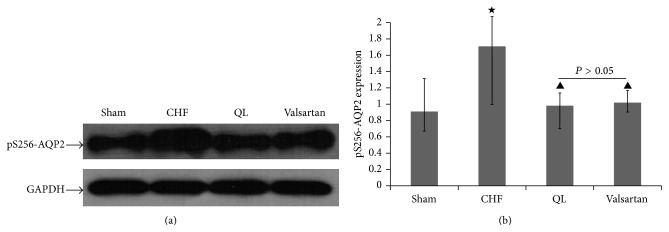
QL reduces renal pS256-AQP2 protein expression in CHF rats. (a) Representative images show that treatment with QL markedly attenuated pS256-AQP2 protein expression in CHF rats. (b) Densitometric analyses of the data demonstrated a significantly increased abundance of AQP2 phosphorylated at ser256 in CHF, rats compared with sham rats. Compared with the CHF rats, the abundance of AQP2 phosphorylated at ser256 was reduced in both QL and Valsartan rats. (^★^ *P* < 0.05, versus the CHF group, ^▲^ *P* < 0.05, versus the sham group).

pS256-AQP2 protein (Figures 6(a) and 6(b)) was significantly upregulated in CHF rats; the level of pS256-AQP2 protein was significantly suppressed not only by valsartan but also by QL treatment.

**Figure 7:**
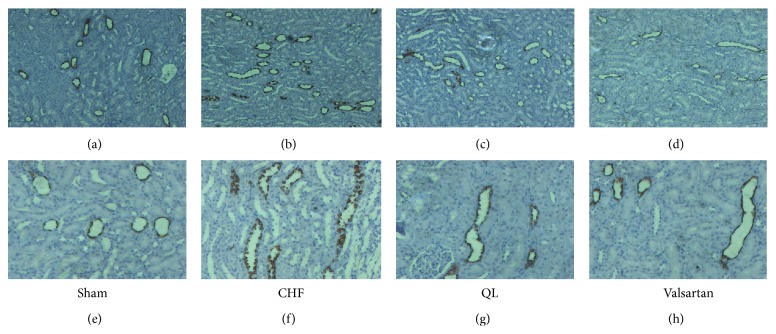
Immunohistochemistry for AQP2 phosphorylated at ser256 in sham ((a) and (e)) and CHF ((b) and (f)), QL ((c) and (g)), and Valsartan ((d) and (h)) at different magnifications. pS256-AQP2 immunoreactivity was detected apically in principal cells in the collecting ducts, and labeling was much stronger in CHF rats than in sham rats, while labeling was much weaker in QL and valsartan rats than in CHF rats.

Consistent with this, immunohistochemistry (Figure 7) showed an increased labeling of pS256-AQP2 at the apical membrane of the IMCD principal cells in CHF animals compared with sham-operated rats. There was a markedly weaker labeling at the apical membrane and a more pronounced intracellular distribution of the protein in rats of QL group and the Valsartan group.

### 3.6. Effects of QL on Plasma AVP and Renal V2R Expression in HF Rats

**Figure 8:**
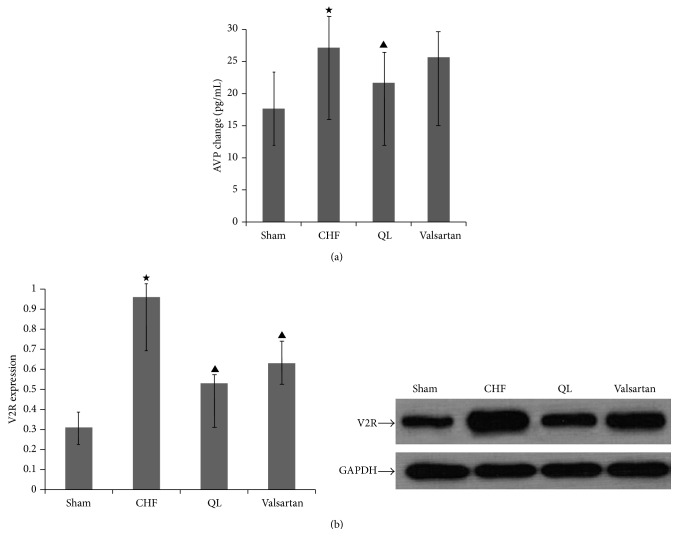
QL reduces the levels of plasma AVP and renal V2R protein expression in CHF rats. (a) AVP levels in plasma were elevated in CHF rats; treatment with QL decreased AVP levels in plasma; however treatment with Valsartan had no effect on plasma AVP in CHF rats. (b) Renal V2R protein increased in CHF rats; treatment with QL significantly attenuated renal V2R protein in CHF rats. (^★^ *P* < 0.05, versus the CHF group, ^▲^ *P* < 0.05, versus the sham group).

To investigate whether QL regulates plasma levels of AVP, we examined plasma AVP levels after treatment for 4 weeks. The levels of AVP in plasma were increased after treatment for 4 weeks in CHF rats. Treatment with QL had minor effect on the attenuation of the elevated levels of AVP in plasma in CHF rats at 4 weeks. In contrast, treatment with Valsartan had no effect on plasma AVP in CHF rats. To determine whether QL could alter the expression of renal V2R, we examined V2R protein expression in medulla at 4 weeks after treatment. We found that CHF rats expressed higher levels of renal V2R than sham rats. After treatment with QL for 4 weeks, V2R protein was significantly decreased (Figures 8(a) and 8(b)).

### 3.7. Effects of QL on Plasma AngII and Renal AT1R Expression in HF Rats

**Figure 9:**
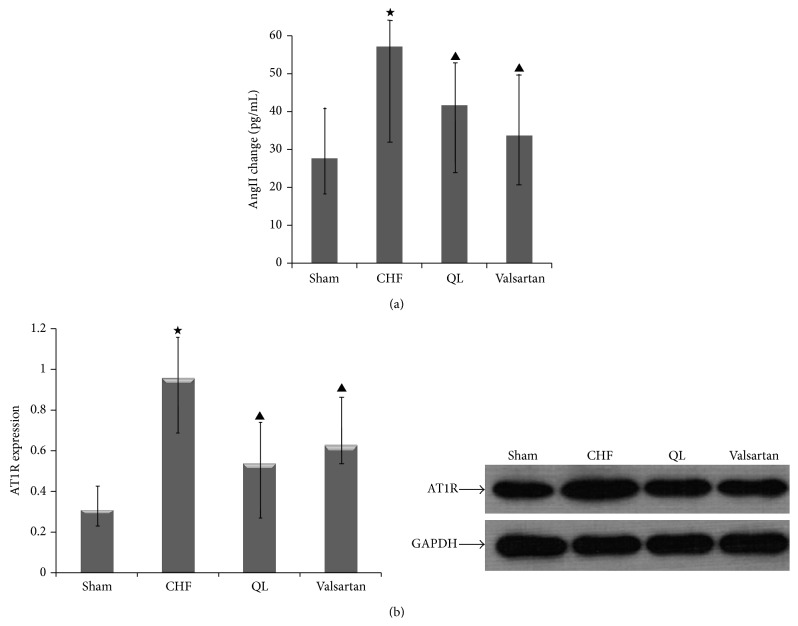
QL reduces the levels of plasma AngII and renal AT1R expression in CHF rats. (a) AngII levels in plasma were elevated in CHF rats; treatment with QL and Valsartan decreased AngII levels in plasma. (b) Renal AT1R protein increased in CHF rats, treatment with QL and Valsartan attenuated renal AT1R protein in CHF rats. (^★^ *P* < 0.05, versus the CHF group, ^▲^ *P* < 0.05, versus the sham group).

To investigate whether QL regulates plasma levels of AngII, we examined plasma AngII levels after treatment for 4 weeks. The levels of AngII in plasma were increased in CHF rats. Treatment with QL and Valsartan had significant effects on the attenuation of the elevated levels of AngII in plasma in CHF rats at 4 weeks. To determine whether QL could alter the expression of renal AT1R, we examined AT1R protein expression in medulla. We found that CHF rats expressed higher levels of renal AT1R than sham rats. After treatment with QL and Valsartan for 4 weeks, AT1R protein was decreased (Figures 9(a) and 9(b)).

## 4. Discussion

We can draw the following conclusions from the present study. (1) QL has diuretic effect. (2) QL also could improve cardiac function in CHF rats. (3) QL could significantly reduce the protein expression of AQP2, pS256-AQP2, V2R, and AT1R in the renal medulla in CHF rats. (4) QL could reduce plasma AVP and AngII level in CHF rats.

From the perspective of traditional Chinese medicine (TCM), the fundamental problem in heart failure is the prolonged deficiency of heart qi and yang, which causes the heart to become too weak to move blood and transport fluid, leading to blood “stasis” and excessive fluid retention [19]. Qili qiangxin capsules are a specific TCM extract obtained from 11 types of herbs, including Ginseng, Radix Astragali, Aconite Root, Salvia miltiorrhiza, Semen Lepidii Apetali, Cortex Periplocae Sepii Radicis, Rhizoma Alismatis, Carthamus tinctorius, Polygonatum Odorati, Seasoned Orange Peel, and Ramulus Cinnamomi, which are well known to have effects on invigorating the heart qi and warming yang, accelerating blood circulation, disinhibiting water, dispersing swelling, and relieving congestion. Pharmacological studies have found that QL contains a number of active substances such as ginseng saponin, astragalus saponin, flavonoid, cardenolide, and phenolic acid which have been proved to have positive inotropic, vasodilation, anti-inflammation, and diuretic effects. In this study, we confirmed that QL treatment has a significant effect to increase urinary output in CHF. QL could also reduce the heart/body weight ratio and the ESV and LVIDs measurements, while it elevates the EF and FS measurements to relieve congestion and improve cardiac function in rats with CHF.

AQP2 is the most important aquaporin, and plays a critical role in chronic heart failure and some diseases [7, 8, 20, 21]. The regulation of AQP2 expression was mainly by AVP signaling. Previous studies demonstrated that circulating AVP levels and V2 receptor mRNA expression are elevated in HF [22, 23]. The binding of AVP to its V2 receptor on the basolateral membrane of principal collecting duct cells initiates a signal transduction cascade that consists of activation of adenylate cyclase via the stimulatory G protein, an increase in intracellular cAMP and intracellular Ca^2+^ levels, and activation of protein kinase A. Subsequently, AQP2 are phosphorylated and translocated from cytosolic storage vesicles to the apical membrane, rendering this membrane permeable to water, thereby increasing water reabsorption. This is called short-term regulation, which occurs within a few minutes after a rise in circulating AVP levels. In the long term, AVP controls AQP2 gene expression through a cAMP response element on the AQP2 promoter [24]. AQP2 contains four serine residues in the C terminal, namely, ser256, ser261, ser264, and ser269. Recent studies demonstrated the view that phosphorylation of ser256 is necessary and sufficient to induce trafficking of AQP2 to the apical membrane [25–27]. In this study, we confirmed that QL treatment significantly reversed the increased protein abundance of AQP2 and phosphorylated AQP2 water channel in the renal medullary collecting duct; immunocytochemistry showed a weaker apical membrane labeling of AQP2 and phosphorylated AQP2 in QL treatment rats compared with model rats. This result suggests that QL exerts its diuretic effect mainly by a mechanism involving suppression of the increased AQP2 trafficking and abundance. At the same time, the result showed that circulating AVP level and V2R protein expression in the renal medulla are decreased in QL treatment rats compared with model rats. In contrast, treatment with Valsartan had no effect on plasma AVP and slight effect on V2R protein expression. This result suggested that QL inhibits free water reabsorption through downregulation of V2R and AQP2 expression. Thus, reducing the effects of AVP and the AVP pathways of V2R-induced upregulation AQP2 and water retention appear to be an attractive therapeutic strategy to promote free water clearance. So far, several vasopressin antagonists are under development. In patients hospitalized with heart failure, oral tolvaptan, a V2 receptor antagonist, improved many, though not all, heart failure signs and symptoms, but without serious adverse events. However, tolvaptan initiated for acute treatment of patients hospitalized with heart failure had no effect on long-term mortality or heart failure-related morbidity [28, 29]. It is possible that QL may have similar therapeutic efficacy to V2 receptor antagonist, and this needs to be addressed in future studies.

In CHF, the renin-angiotensin-aldosterone system has also been demonstrated to play a critical role in the regulation of renal sodium and water metabolism through a variety of physiological pathways. In particular, AngII has known effects on the regulation of renal hemodynamics, glomerular filtration rate. Moreover, several recent studies have demonstrated that angiotensin II could play a role in the regulation of renal water reabsorption by changing intracellular AQP2 targeting and/or AQP2 abundance through inducing vasopressin V2-receptor mRNA expression, in addition to the AVP [30]. This potentiated the effects of vasopressin-modulated AQP2 trafficking and expression [9]. Blockade of the AngII AT1 receptor in rats cotreated with dDAVP and dietary NaCl-restriction (to induce high plasma endogenous AngII) resulted in an increase in urine production and blunted the AVP-induced upregulation of AQP2 [11]. AT1R blocker candesartan has been shown to decrease increased apical targeting of AQP2 and p-AQP2 in inner medulla of HF [8]. Thus, inhibiting AngII and the AngII pathways of AT1R-induced upregulation of AQP2 and water retention may be another important pathway to promote water clearance. Our study proved that QL and another type AT1R blocker Valsartan had the similar effects. Meanwhile, the result showed that plasma AngII level and AT1R protein expression in the renal medulla are decreased in treatment rats compared with model rats, suggesting that QL inhibits water reabsorption through downregulation of AT1R and AQP2 expression.

## 5. Conclusion

In conclusion, the present study provided evidences for the 4 weeks use of QL for diuretics and cardiac function improvement in CHF rat model. Based on our results, we conclude that QL carries out its effects mainly by reversing abundance AQP2 and pS256-AQP2 protein as well as redistribution of AQP2 and pS256-AQP2 from apical to intracellular domains. The possible mechanisms may involve inhibition of V2R and AT1R. However, Valsartan carries out its effects mainly by AngII signaling regulating. Therefore, QL has multiple effects on chronic heart failure. Further studies should explore the deeper mechanism through which QL improves water metabolism to ultimately offer new avenues for the prevention and treatment of this and other related diseases.

## Figures and Tables

**Table 1:** Echocardiographic ejection fraction level in different groups before treatment with qili qiangxin ($\overset{-}{x}\;\pm \;s$).

Group	*n*	EF (%)
Sham	10	84.490 ± 7.3354
CHF	12	44.708 ± 8.4369^*^
QL	9	47.933 ± 9.1211^*^
Valsartan	9	47.911 ± 9.1068^*^

^*^ *P* > 0.05.
